# Commentary: Anticoagulant outcomes in managing tumor thrombus: a systematic review

**DOI:** 10.3389/fonc.2026.1820318

**Published:** 2026-05-08

**Authors:** Elena-Mihaela Cordeanu, Dominique Stephan

**Affiliations:** 1 Department of Hypertension and Vascular Diseases, Clinical Pharmacology, Strasbourg University Hospitals, Strasbourg, France; 2 Translational Cardiovascular Medicine, UR 3074, CRBS, University of Strasbourg, Strasbourg, France

**Keywords:** anticoagulant, blant thrombus, hepatocellualr carcinoma, renal cell carcinoma, tumor thrombus, venous thromboembolism

## Introduction

The systematic review by Alfehaid et al. provides a comprehensive synthesis of evidence on anticoagulation in solid tumor-associated tumor thrombus (TT) (1). The authors demonstrate that anticoagulation alone does not induce TT regression, fails to reduce thromboembolic recurrence, and carries significant bleeding risk, particularly in renal cell carcinoma (RCC). However, we believe that several underexplored dimensions warrant further discussion, particularly regarding the imaging-guided stratification of thrombus composition, the distinction between prophylactic and therapeutic anticoagulation intent, and the emerging influence of modern systemic therapies on TT biology.

## The “mixed thrombus” blind spot

A central finding of this review is that TT, composed predominantly of viable malignant cells, is intrinsically resistant to anticoagulants. However, the clinical reality is that TT and bland thrombus frequently coexist within the same vascular territory. In a retrospective cohort of 211 patients with TT, 21.8% had concurrent venous thromboembolism (VTE) at the time of TT diagnosis, and the majority involved the same vascular territory (2). Since anticoagulation can only act on the fibrin-rich bland component, treatment decisions hinge on accurately distinguishing both components. Alfehaid et al. acknowledge that subgroup outcomes for patients with mixed thrombotic components were not systematically evaluated, and thromboembolic events were not adjudicated as bland versus malignant. This coexistence is biologically predictable. Mechanistically, TT formation involves heparanase-mediated endothelial necroptosis, while platelet-tumor microaggregate formation, and neutrophil extracellular trap (NET) deposition, further generate prothrombotic milieu that may promote superimposed bland thrombus (3).

Consistently, single-cell transcriptomic data demonstrate that TT in RCC is a highly organized multicellular ecosystem containing macrophages, malignant cells, endothelial cells, and myofibroblasts within a remodelled extracellular matrix (4). These findings reinforce the plausibility of anticoagulant inefficacy in dissolving an integrated tumor-stromal tissue.

Differentiating tumor from bland thrombus on imaging remains imperfect. While tumor contiguity, vessel expansion, arterial enhancement, neovascularization, and restricted diffusion on MRI are well-established features of TT (5, 6), these criteria may fail when both components coexist, particularly in small-caliber vessels, or in the setting of portal hypertension where hemodynamic alterations confound imaging interpretation (7). Existing noninvasive diagnostic criteria, such as the A-VENA score and SUVmax thresholds on ^18^F-FDG PET/CT, have been validated only for binary classification as malignant or benign, but none addresses mixed thrombus quantification (8). Contrast-enhanced ultrasound (CEUS), which demonstrates high sensitivity and specificity in the portal venous system, has not been evaluated for quantifying the mixed malignant/bland thrombus burden, nor have radiomics-based approaches been applied to this specific clinical question (7).

We suggest that future research should prioritize the development and validation of imaging-based criteria to quantify the relative malignant versus bland composition of venous thrombi. Such stratification could identify a subgroup of patients with predominantly bland thrombus superimposed on TT who might benefit from anticoagulation, while sparing patients with pure TT from unnecessary bleeding risk.

## Prophylactic versus therapeutic intent: an unresolved question

Alfehaid et al. appropriately note that most included studies evaluated therapeutic-dose anticoagulation and that data on thromboprophylaxis in TT patients are sparse. Patients with TT harbor all components of Virchow’s triad (endothelial disruption at the tumor-vessel interface, stasis from venous obstruction, and tumor-induced hypercoagulability), placing them at high risk for secondary bland thrombus formation and systemic VTE. In hepatocellular carcinoma (HCC) specifically, portal hypertension and reduced portal flow velocity create hemodynamic conditions that independently favor bland thrombus accretion, beyond the malignant component alone (2). Portal vein TT prevalence in HCC is likely underestimated, as TT is found incidentally in 14% of liver biopsies and up to 62% of autopsied livers in historical series, suggesting that subclinical mixed thrombus is far more common than imaging-based studies indicate (8). In RCC, VTE incidence is estimated at 7.4% when TT is confined to the renal vein, rising to 22% with IVC involvement below the diaphragm and 55.3% above it (9, 10). This gradient suggests that prophylactic anticoagulation strategies should be tailored not only to thrombus phenotype but also to the anatomical extent of vascular invasion, a dimension absent from the studies reviewed by Alfehaid et al. The question of whether prophylactic anticoagulation can prevent bland thrombus accretion on TT surfaces without the bleeding burden of therapeutic dosing remains unanswered and represents, in our view, the most clinically relevant and feasible research question in this field.

## Systemic therapies and the evolving biology of TT

Finally, the review correctly mentions that modern systemic therapies, including immune checkpoint inhibitors and tyrosine kinase inhibitors, may induce TT regression independently of anticoagulation. Single-cell transcriptomic profiling has revealed that TT in RCC harbors a higher proportion of tissue-resident CD8+ T cells in a progenitor exhausted state compared with matched primary tumors (4). This T cell subset has been linked to favorable responses to anti-PD-1 therapy in advanced RCC, providing a mechanistic rationale for the TT regressions reported with neoadjuvant immunotherapy (4). However, VEGF-targeting therapies, including bevacizumab-based combinations and lenvatinib, have been associated with TT regression but also increased hemorrhagic risk, creating a challenging risk-benefit equation (3, 8, 11). Whether concurrent anticoagulation modulates these treatment effects, positively or negatively, remains unknown. Prospective registries capturing anticoagulation exposure alongside tumor-directed therapies in patients with well-characterized TT would be invaluable to disentangle these competing effects.

## Conclusion and perspectives

**Figure 1:**
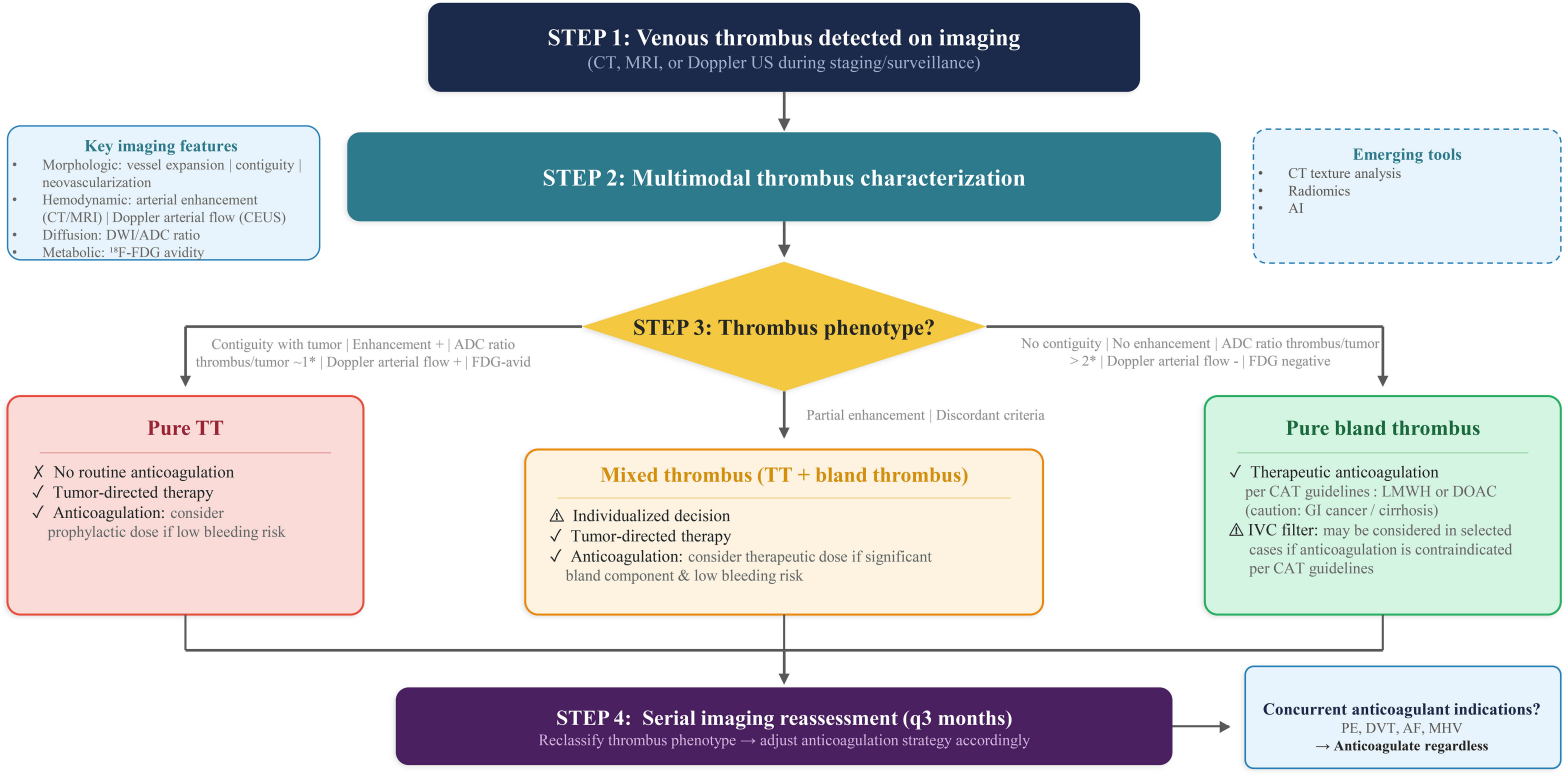
Proposed imaging-guided algorithm for anticoagulation decision-making in cancer patients with venous tumor thrombus (portal vein, renal vein, inferior vena cava). *ADC ratio thresholds are derived from a single portal vein study (6) and require validation across vascular territories and tumor types. ADC, apparent diffusion coefficient; AF, atrial fibrillation; CAT, cancer-associated thrombosis; CE-CT, contrast-enhanced computed tomography; CEUS, contrast-enhanced ultrasound; DOAC, direct oral anticoagulant; DVT, deep vein thrombosis; DWI, diffusion-weighted imaging; IVC, inferior vena cava; LMWH, low-molecular-weight heparin; MHV, mechanical heart valve; MRI, magnetic resonance imaging; PE, pulmonary embolism; TT, tumor thrombus.

In summary, the work of Alfehaid et al. establishes that routine anticoagulation for isolated TT is not supported by current evidence. We propose an imaging-guided algorithm (**Figure 1**) for case-by-case anticoagulation decisions and serial imaging monitoring for isolated TT. Our approach incorporates multimodal thrombus phenotyping as the central decision node, enabling a more systematic stratification between pure TT, mixed thrombus, and pure bland thrombus, essential for future prospective study design. Building on this foundation, we propose that three priorities should guide future research: (a) development of imaging-based tools to characterize mixed malignant/bland thrombus composition; (b) prospective evaluation of prophylactic versus therapeutic anticoagulation strategies, stratified by thrombus phenotype; and (c) integration of anticoagulation data within trials of anticancer systemic therapies. Addressing these gaps will be essential to move from the current empirical, one-size-fits-all approach toward a precision-based management of TT in cardio-oncology.
